# Phospholipase D from *Loxosceles* *laeta* Spider Venom Induces IL-6, IL-8, CXCL1/GRO-α, and CCL2/MCP-1 Production in Human Skin Fibroblasts and Stimulates Monocytes Migration

**DOI:** 10.3390/toxins9040125

**Published:** 2017-04-05

**Authors:** José M. Rojas, Tomás Arán-Sekul, Emmanuel Cortés, Romina Jaldín, Kely Ordenes, Patricio R. Orrego, Jorge González, Jorge E. Araya, Alejandro Catalán

**Affiliations:** 1 Laboratorio de Parasitología Molecular, Departamento de Tecnología Médica, Facultad de Ciencias de la Salud, Universidad de Antofagasta, Antofagasta, CP 1270300, Chile; jose.rojas@uantof.cl (J.M.R.); tomas.aran@uantof.cl (T.-A.S.); emmanuel_cr@hotmail.cl (E.C.); romyjaldin@gmail.com (R.J.); kely.ordenes@uantof.cl (K.O.); jorge.gonzalez@uantof.cl (J.G.); jearoj@yahoo.com (J.E.A.); 2 Departamento Biomédico, Facultad de Ciencias de la Salud, Universidad de Antofagasta, Antofagasta, CP 1270300, Chile; patricio.orrego@uantof.cl

**Keywords:** phospholipase D, *Loxosceles laeta*, monocytes migration, chemokines

## Abstract

Cutaneous loxoscelism envenomation by *Loxosceles* spiders is characterized by the development of a dermonecrotic lesion, strong inflammatory response, the production of pro-inflammatory mediators, and leukocyte migration to the bite site. The role of phospholipase D (PLD) from *Loxosceles* in the recruitment and migration of monocytes to the envenomation site has not yet been described. This study reports on the expression and production profiles of cytokines and chemokines in human skin fibroblasts treated with catalytically active and inactive recombinant PLDs from *Loxosceles laeta* (rLlPLD) and lipid inflammatory mediators ceramide 1-phosphate (C1P) and lysophosphatidic acid (LPA), and the evaluation of their roles in monocyte migration. Recombinant rLlPLD1 (active) and rLlPLD2 (inactive) isoforms induce interleukin (IL)-6, IL-8, CXCL1/GRO-α, and CCL2/monocyte chemoattractant protein-1 (MCP-1) expression and secretion in fibroblasts. Meanwhile, C1P and LPA only exhibited a minor effect on the expression and secretion of these cytokines and chemokines. Moreover, neutralization of both enzymes with anti-rLlPLD1 antibodies completely inhibited the secretion of these cytokines and chemokines. Importantly, conditioned media from fibroblasts, treated with rLlPLDs, stimulated the transmigration of THP-1 monocytes. Our data demonstrate the direct role of PLDs in chemotactic mediator synthesis for monocytes in human skin fibroblasts and indicate that inflammatory processes play an important role during loxoscelism.

## 1. Introduction

Envenomation from *Loxosceles* spider bites produces a clinical picture known as loxoscelism. This genus belongs to the *Sicariidae* family, and includes a large number of species described and extensively distributed worldwide [1]. However, the only species from genus *Loxosceles* routinely involved in human poisoning are *Loxosceles laeta*, *Loxosceles intermedia*, *Loxosceles rufescens*, *Loxosceles gaucho*, and *Loxosceles reclusa* [2,3,4]. In Chile, it appears that *L. laeta* is the only relevant species [2,5] that produces venom with strong biological activities [6].

Loxoscelism occurs in two clearly defined clinical forms: cutaneous loxoscelism and viscero-cutaneous or systemic loxoscelism. Cutaneous loxoscelism is the most frequently occurring clinical form, and is characterized by severe skin alterations such as erythema, edema, and a marked inflammatory response, followed by the development of a dermonecrotic lesion with the typical presence of a livedoid plate. Conversely, systemic loxoscelism is a more severe but less common clinical picture characterized by intravascular hemolysis of erythrocytes, vascular alterations, and renal insufficiency, sometimes leading to patient death [7,8]. *Loxosceles* spider venom is mainly comprised of the phospholipase D (PLD) enzyme family [4], which have been identified as the agent responsible for loxoscelism’s tissue damage, involving alterations such as plaque aggregation [9], dermonecrosis [8,10], complement-dependent hemolysis of erythrocytes [10,11,12,13], damage to blood vessels and fibrinogenolysis [14], and deregulation of neutrophil activation, which depends on endothelial cells [15]. The *Loxosceles* PLDs can hydrolyze sphingomyelin to form ceramide 1-phosphate (C1P) and choline [9,10,11]. It can also hydrolyze lysophospholipids such as lysophosphatidylcholine to form lysophosphatidic acid (LPA) and choline [16,17,18].

Regardless of the severity of the clinical form, one of the main features of *Loxosceles* venom poisoning is the strong inflammatory reaction in the bite site dermis. This is characterized by a massive infiltration of inflammatory cells, which can persist for weeks to months after the venom contact [19]. Thus, mice injected with *L. gaucho* venom exhibited increased macrophages and neutrophils as soon as 30 min post-injection and increasing until 24 h, accompanied by release of inflammatory mediators such as interleukin-6 (IL-6), monocyte chemoattractant protein-1 (MCP-1), and keratinocyte chemoattractant (KC) [20]. However, the role of *Loxosceles* PLDs in the inflammatory response and in leukocyte recruitment is still controversial and not fully understood. In this regard, *Loxosceles* venom has no reported direct stimulatory effect on leukocytes [21]. Instead, leukocyte activation involves blood vessel endothelial cells, which indirectly affect leukocytes when exposed to the venom. In this case, the venom acts as a potent endothelial cell agonist, inducing surface E-selectin expression and secretion of granulocyte macrophage colony-stimulating factor (GM-CSF) and interleukin-8 (IL-8/CXCL8) [15]. Then, the neutrophils migrate to the bite site and are subsequently activated and degranulate, resulting in inflammation and necrosis [15]. Additionally, dermis fibroblasts, together with the endothelia, have been suggested to be involved in the deregulated leukocyte activation, since up-regulated expression of IL-6, IL-8, CXCL1, and CXCL2 has been reported in human foreskin fibroblast Hs68 cells exposed to recombinant *L. reclusa* PLD [22]. Thus, the product of sphingomyelin hydrolysis (C1P [22]) or the product of lysophosphatidylcholine (LPC) hydrolysis (LPA [23]) could reportedly be triggering the expression of these inflammatory mediators and neutrophil infiltration. Some recent results support this mechanism, including implications that LPA produced by LPC from *L. similis* venom or *L. intermedia* SMD could mediate the cytokine and chemokine release in fibroblasts, and that this effect is mediated by an LPA receptor [24]. Thus, the cytokines and chemokines produced by endothelial cells and fibroblasts in response to *Loxosceles* venom strongly suggest that these proteins play key roles in the leukocyte recruitment to the injury site and the development of inflammatory responses during poisoning. Most recent studies have focused on the chemotactic mediators induced by the *Loxosceles* venom on in vivo neutrophils recruitment. However, despite the importance of monocytes/macrophages in the inflammatory response, there are no studies demonstrating the link between cytokine and chemokine release in human skin fibroblasts in response to PLDs from *Loxosceles* venom and monocytes recruitment to the site of injury during cutaneous loxoscelism.

Previously, we expressed and purified two phospholipase D (PLD) isoforms of the spider *Loxosceles laeta (rLIPLD1 and rLIPLD2)*. The recombinant protein rLIPLD1 had hydrolytic activity on sphingomyelin and in vitro hemolytic activity on human red blood cells, whereas rLIPLD2 was inactive. This latter corresponds to a truncated isoform lacking one of the two essential histidine residues implicated in the catalytic action of the enzyme [25]. We used these recombinant PLD proteins with different activity as a tool to evaluate the inflammatory cytokine and chemokine expression and production profiles in human skin fibroblasts treated with recombinant PLDs of *L. laeta*, compared to C1P and LPA, and to determine their role in monocyte cells migration. Our data develop the expression and production profiles of cytokines and chemokines in human skin fibroblasts treated with recombinant PLDs of *L. laeta* (rLlPLDs) and the lipid inflammatory mediators C1P and LPA. The production of IL-6, IL-8, CXCL1, and CCL2 was found to be a direct consequence of PLD’s effect of *L. laeta* on fibroblast, while C1P and LPA only had minor effects on the secretion of these cytokines/chemokines. Finally, our data demonstrate that these chemotactic mediators promote monocyte migration.

## 2. Results

### 2.1. Cytokines/Chemokines Profile in Human Skin Fibroblasts in Response to Recombinant PLD from *L. laeta* Venom

The human skin fibroblast HFF-1 cell line was used as a model for this study. These cells were treated with recombinant PLD isoforms from *L. laeta* (rLlPLD1 or rLlPLD2), which have been described previously [25]. In addition, cells were treated with the bioactive lipids C1P and LPA, which correspond, respectively, to the main products generated from the hydrolysis of sphingomyelin and lysophosphatidylcholine by *Loxosceles* PLDs [11,18]. Each treatment was evaluated to determine its cytotoxicity in skin fibroblasts after 24 h. As expected, rLlPLD1 demonstrated cytotoxicity (death >50%) for fibroblasts at concentrations higher than 15 μg/mL (IC50 = 15.8 μg/mL). The remaining treatments (C1P, LPA, and rLlPLD2) exhibited no cytotoxicity (about 100% viability) on the cells at any of the evaluated concentrations.

**Figure 1:**
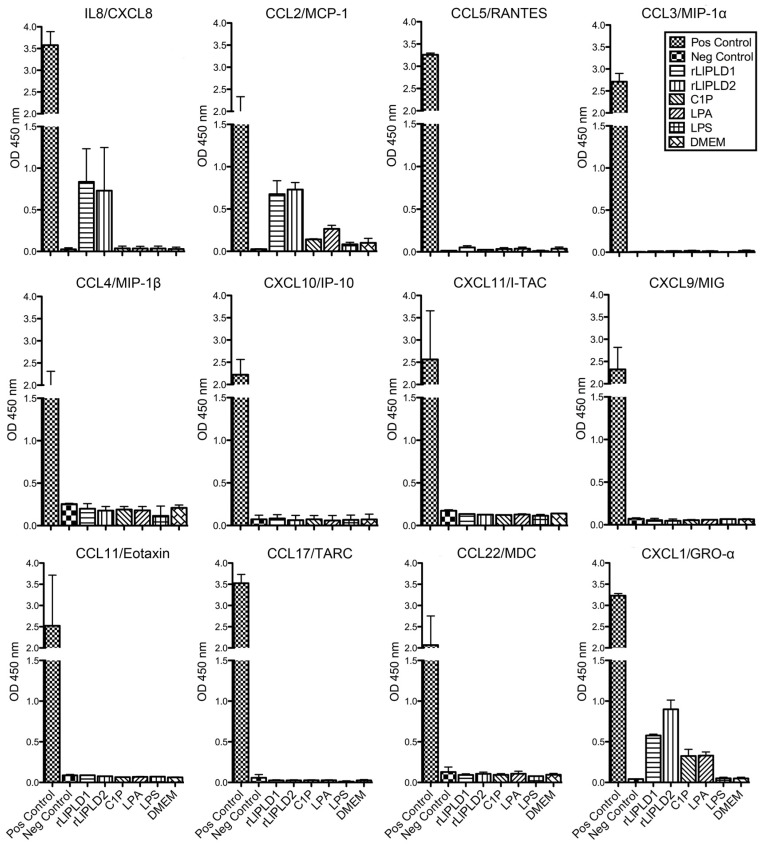
Chemokine ELISArray profiles of human skin fibroblasts HFF-1 cells. Fibroblasts HFF-1 (1 × 10^5^ cells/mL) cultures were treated with 5 μg/mL recombinant PLD of *L. laeta* (rLlPLD1, active) or (rLlPLD2, inactive), 10 μM ceramide 1-phosphate (C1P), 10 μM lysophosphatidic acid (LPA), or 10 μg/mL LPS and incubated for 24 h at 37 °C in an atmosphere containing 5% CO_2_. Dulbecco’s Modified Eagle’s Medium (DMEM) without fetal bovine serum (FBS) was used as control. Culture supernatants were recovered and used for chemokine screening in a Common Chemokines Multi-Analyte ELISArray kit (QIAGEN) panel for the detection of interleukin (IL)-8, monocyte chemoattractant protein-1 (MCP-1), RANTES, MIP-1α, MIP-1β, IP-10, I-TAC, MIG, Eotaxin, TARC, MDC, and GRO-α, according to the manufacturer’s instructions. Absorbance at 450 nm was measured in a micro-plate reader, and the chemokine levels were determined in relation to the absorbance value of the negative control (assay buffer) and compared to the positive control (containing standard of all 12 chemokines). The presence of chemokines in culture supernatants was considered for absorbance values over negative control. Results were expressed as mean ± SEM of two experiments performed independently.

Then, we screened for the synthesis and secretion profile of inflammatory cytokines produced in fibroblasts treated with recombinant PLDs (rLlPLD1 and rLlPLD2) at 5 μg/mL, and also with C1P (10 μM), LPA (10 μM), or LPS (10 μg/mL) using a 12 multi-analyte ELISArray panel. An inflammatory cytokines panel (IL1-α, IL1-β, IL2, IL4, IL6, IL8, IL10, IL12, IL17A, interferon (IFN)-γ, TNF-α, and GM-CSF), and a chemokines panel (IL8, MCP-1, RANTES, MIP-1α, MIP-1β, IP-10, I-TAC, MIG, Eotaxin, TARC, MDC, and GRO-α) were evaluated. The panel of 12 inflammatory cytokines showed IL-8 presence and at a lower level, IL-6 in the supernatants of fibroblasts treated with rLlPLDs as well as those treated with bioactive lipids C1P and LPA (Supplementary Materials Figure S1). Meanwhile, the remaining cytokines were not produced. Conversely, the 12-chemokine profiles indicated that IL-8, CCL2/MCP-1, and CXCL1/GRO-α, were only secreted in fibroblasts’ supernatant when they were treated with rLlPLD1 or rLlPLD2; meanwhile, C1P and LPA only induced secretion of CCL2 and CXCL1, but not IL-8 (Figure 1). Fibroblasts treated with LPS did not secrete cytokines or chemokine.

### 2.2. Recombinant PLD from *L. laeta* Induce Expression and Secretion of IL-6, IL-8, CXCL1/GRO-α, and CCL2/MCP-1 in Human Skin Fibroblasts

**Figure 2:**
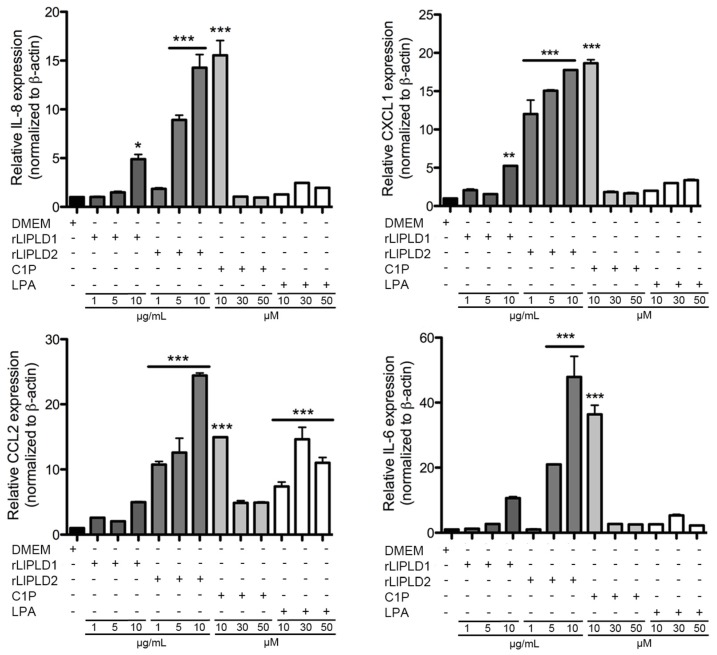
Quantitative reverse transcription PCR (RT-qPCR) for the expression of cytokines and chemokines in human skin fibroblast HFF-1 cells treated with PLDs of *L. laeta* at 6 h. Fibroblasts HFF-1 cells were incubated for 6 h at 37 °C in an atmosphere containing 5% CO_2_ with DMEM containing no FBS in the presence of different concentrations of rLlPLD1, rLlPLD2, C1P, and LPA. A basal expression condition was evaluated in cell cultures treated with only DMEM. cDNA was synthesized from mRNA taken from the supernatant in each experiment and amplified by RT-qPCR. The human β-actin gene was used as the reference gene. Differences in the relative expression of human genes IL-6, IL-8, CXCL1, and CCL2 mRNA expression for each gene were standardized using the human β-actin gene expression levels as a reference gene, and results are presented as the average relative expression over control ± SEM of two experiments performed in duplicate.

The mRNA expression pattern for genes IL-6, IL-8, CXCL1/GRO-α, and CCL2/MCP-1 were then evaluated. The study was performed in skin fibroblasts under basal conditions (Dulbecco’s Modified Eagle’s Medium (DMEM) only) as a control and with increasing concentrations of rLlPLD1 or rLlPLD2. Additionally, fibroblast cultures were incubated with different concentrations of C1P or LPA. Quantitative reverse transcription PCR (RT-qPCR) performed on fibroblast cDNA obtained after 6 and 24 h of treatment indicated low constitutive expression levels of CCL2, IL-6, and IL-8 mRNA. The recombinant proteins rLlPLD1 and rLlPLD2 showed a differential induction of IL-6, IL-8, CXCL1, and CCL2 mRNA expression in fibroblasts at 6 h (Figure 2) and 24 h (Figure S2). At 6 h of treatment, rLlPLD2 induced a marked expression of cytokines and chemokines compared to rLlPLD1 (Figure 2). However, this effect reverted at 24 h of treatment, where the expression of IL-8, CXCL1, and CCL2 decayed markedly for rLlPLD2 and increased for treatment with rLlPLD1 (Figure S2). The latter may indicate an early effect of rLlPLD2 on the induction of expression of these chemokines, and a late effect of rLlPLD1. On the other hand, IL-6 expression dropped dramatically at 24 h of treatment (Figure S2), while C1P and LPA induced a markedly expression of CCL2 and a low expression of CXCL1, IL-6, and IL-8 at 6 h, which decayed at 24 h of treatment. C1P also induced a markedly expression of IL-6, IL-8, CXCL1, and CCL2 at 6 h of treatment at a concentration of 10 μM, but not at higher concentrations.

**Figure 3:**
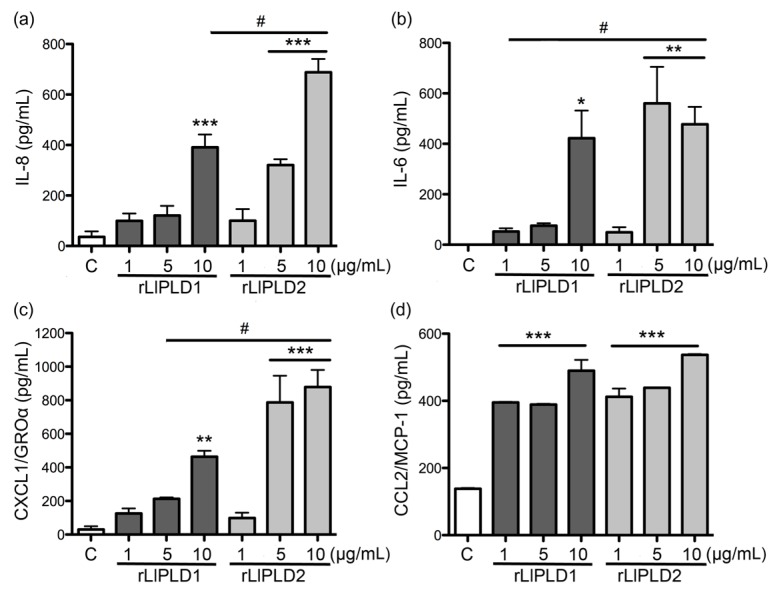
ELISA quantitation of IL-6, IL-8, CXCL1/GROα, and CCL2/MCP-1 produced by human skin fibroblasts HFF-1 cells treated with PLDs of *L. laeta*. Fibroblast HFF-1 cells (1 × 10^5^ cells/mL) cultures were treated with 1, 5, and 10 μg/mL of rLlPLD1 or rLlPLD2 in DMEM serum-free medium and incubated for 24 h at 37 °C in an atmosphere containing 5% CO_2_. DMEM serum-free medium without treatment was used as a negative control. The supernatant from each experiment was recovered and evaluated with ELISA for (**a**) IL-8; (**b**) IL-6; (**c**) CXCL1; and (**d**) CCL2 production. Absorbance at 450 nm was measured, and the levels of IL-6, IL-8, CXCL1, and CCL2 (pg/mL) were determined from a standard calibration curve at known concentrations. Three independent experiments were performed, and results are expressed as mean ± SEM of duplicates. Significance was evaluated with an ANOVA one-way with Bonferroni post-hoc test; (*) indicates *p* < 0.05, (**) indicates *p* < 0.01, (***) indicates *p* < 0.001. # Indicates statistical significance with *p* < 0.05 between rLlPLD1 and rLlPLD2.

**Figure 4:**
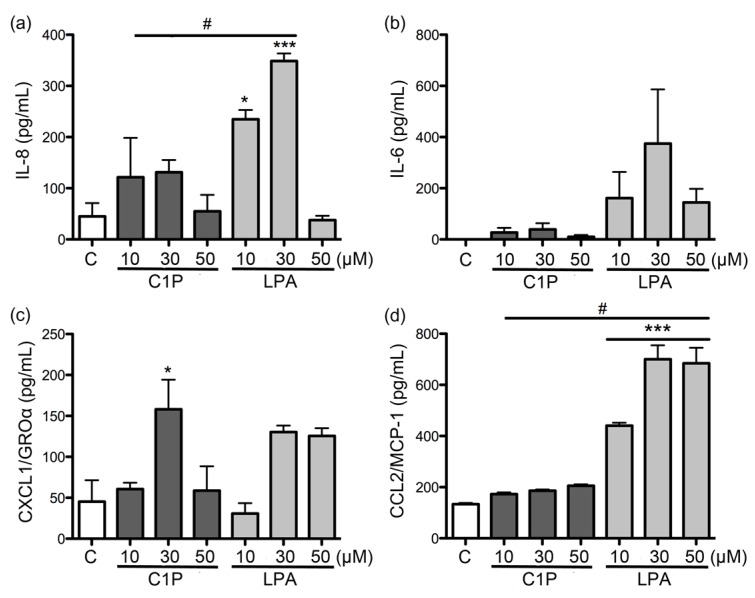
ELISA quantitation of IL-6, IL-8, CXCL1/GROα, and CCL2/MCP-1 produced by human skin fibroblasts HFF-1 cells treated with C1P and LPA. Fibroblast HFF-1 cell (1 × 10^5^ cells/mL) cultures were treated with 10, 30, and 50 μM of C1P or LPA in DMEM serum-free medium and incubated for 24 h at 37 °C in an atmosphere containing 5% CO_2_. DMEM serum-free medium without treatment was used as a negative control. The supernatant of medium was recovered and evaluated by ELISA for (**a**) IL-8; (**b**) IL-6; (**c**) CXCL1; and (**d**) CCL2 production. Absorbance at 450 nm was measured, and the levels of IL-6, IL-8, CXCL1, and CCL2 (pg/mL) were determined from a standard calibration curve at known concentrations. Three independent experiments were performed, and results are expressed as mean ± SEM of duplicates. Significance was evaluated with an ANOVA one-way with Bonferroni post-hoc test; (*) indicates *p* < 0.05, (**) indicates *p* < 0.01, (***) indicates *p* < 0.001. # Indicates statistical significance with *p* < 0.05 between C1P and LPA.

IL-6, IL-8, CXCL1, and CCL2 concentrations were evaluated by ELISA to determine if changes in mRNAs expression of these cytokines and chemokines were accompanied by an increase in their secretion in the supernatant of fibroblast culture. rLlPLD1 and rLlPLD2 were observed to induce markedly increased IL-6, IL-8, CCL2, and CXCL1 secretion in fibroblasts. However, the rLlPLD2 effect on cytokine and chemokine synthesis was significantly stronger than that of rLlPLD1. In fact, the IL-8 secretion induced by rLlPLD2 was 2.5 and 1.7 times greater at concentrations of 5 μg/mL and 10 μg/mL, respectively, than secretion induced by rLlPLD1 (Figure 3a). In addition, under the same experimental conditions, IL-6 secretion induced by rLlPLD2 was 7.5 times higher than from rLlPLD1 when a concentration of 5 μg/mL was used (Figure 3b). The effect of rLlPLD2 for CXCL1 was 3.7 and 1.8 times greater than rLlPLD1 at concentrations of 5 μg/mL and 10 μg/mL, respectively (Figure 3c). However, CCL2 secretion was similar after treatment with both recombinant proteins (Figure 3d). Additionally, the effects of C1P and LPA on IL-6, IL-8, CXCL1, and CCL2 secretion in fibroblast culture supernatants were evaluated. Figure 4 shows that LPA affected IL-8 secretion at a rate of 2.7 times greater than C1P at concentrations of 30 μM (Figure 4a). LPA, but not C1P, had an effect on the secretion of IL-6 (Figure 4b). Both C1P and LPA had a similar effect on CXCL1 secretion (Figure 4c). The effect of LPA on secretion of CCL2 was 3.3 and 3.8 times greater than C1P at concentrations of 10 μM and 30 μM, respectively (Figure 4d). The average concentrations of cytokines and chemokines induced by C1P or LPA in the supernatant of fibroblasts were lower than those induced by treatment with either recombinant PLD of *L. laeta* under all conditions tested.

### 2.3. Cytokines/Chemokines Secreted by Fibroblasts Treated with PLDs from *L. laeta* Promote Monocyte Migration

**Figure 5:**
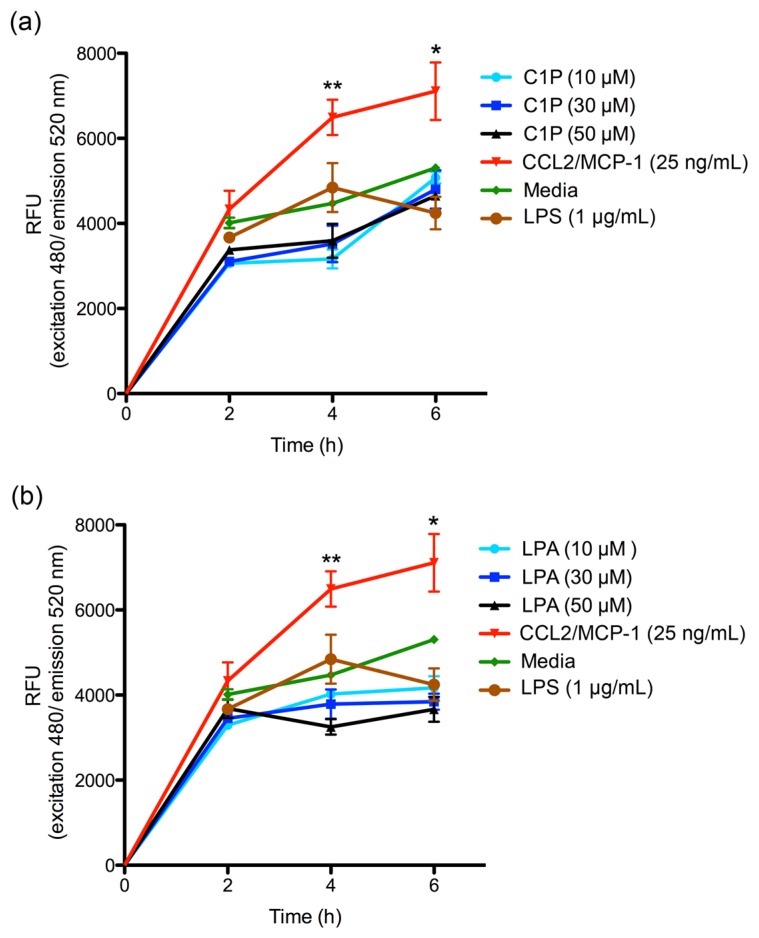
THP-1 monocytes migration assay. THP-1 monocytes were evaluated in a migration assay using the CytoSelect™ 96-Well Migration Assay kit at 2, 4, and 6 h according to manufacturer’s instructions. A concentration of 1 × 10^5^ cells/mL of THP-1 monocytes in RPMI 1640 serum-free medium were incubated with: (**a**) C1P at concentration of 10, 30, and 50 µM; and (**b**) LPA at concentrations of 10, 30, and 50 µM. Cells that had transmigrated to the lower chamber were disassociated from the membrane and lysed to be quantified using CyQuant^®^ GR Fluorescent Dye; fluorescence was read at excitation 480 nm/emission 520 nm and expressed as RFU. Migration negative control was RPMI 1640 serum-free medium. Human recombinant CCL2/MCP-1 at 25 ng/mL was used as a positive control. Three independent experiments were performed, and results are expressed as mean ± SEM of duplicates. Significance was evaluated using a one-way ANOVA with Bonferroni post-hoc test; (*) indicates *p* < 0.05, (**) indicates *p* < 0.01.

Cell migration assays using THP-1 monocyte cultures were performed to evaluate the biological relevance of IL-6, IL-8, CXCL1, and CCL2 secreted by human skin fibroblasts on leukocyte migration. THP-1 monocyte cultures were therefore incubated in different chemoattractant media, and their ability to migrate through a 5 μm pore size in a transmigration chamber was evaluated. First, the chemoattractant ability of C1P or LPA at different concentrations was evaluated for THP-1 monocytes. Both C1P (Figure 5a) and LPA (Figure 5b) were found to be unable to stimulate by themselves the migration of THP-1 monocytes over the range of times and concentrations tested.

**Figure 6:**
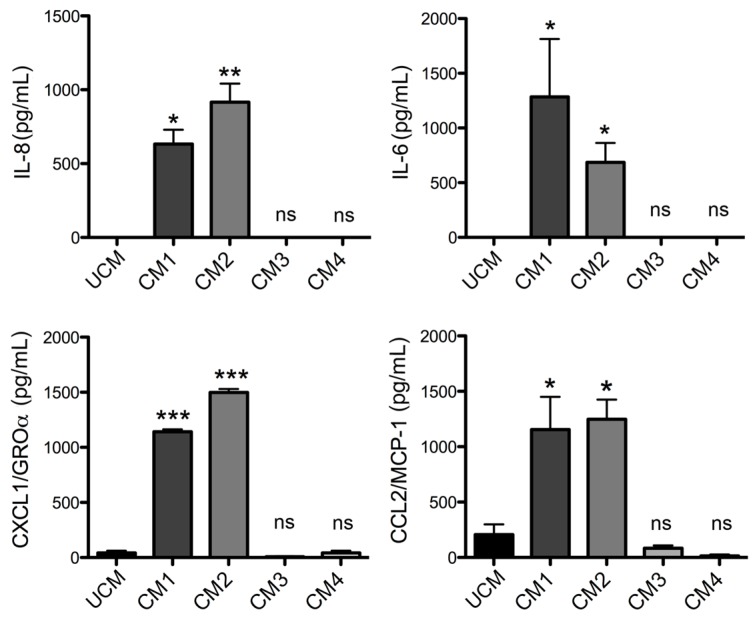
ELISA quantitation of IL-6, IL-8, CXCL1, and CCL2 in conditioned media of fibroblast HFF-1 cells. Fibroblast HFF-1 cells were incubated at 37 °C for 24 h in an atmosphere containing 5% CO_2_ in incomplete DMEM medium containing 5 μg/mL rLlPLD1 (Conditioned Medium 1; CM1), 5 μg/mL rLlPLD2 (Conditioned Medium 2; CM2), or with recombinant rLlPLD1 and rLlPLD2 neutralized with rabbit polyclonal antibody for rLlPLD1 at a concentration of 5 μg/mL in DMEM medium; neutralized rLlPLD1 (Conditioned Medium 3; CM3), and neutralized rLlPLD2 (Conditioned Medium 4; CM4). These were also compared to cells grown in untreated incomplete DMEM medium (Unconditioned Medium, UCM). Then, the cell culture supernatant was recovered and evaluated using ELISA. Absorbance at 450 nm was measured, and the levels of IL-6, IL-8, CXCL1, and CCL2 (pg/mL) were determined for a standard calibration curve at known concentrations. Three independent experiments were performed, and results are expressed as mean ± SEM of duplicates. (*) Indicates *p* < 0.05, (**) indicates *p* < 0.01, (***) indicates *p* < 0.001.

Subsequently the ability of the supernatant of fibroblasts treated with rLlPLD1 (Conditioned Medium 1; CM1) or rLlPLD2 (Conditioned Medium 2; CM2) to stimulate the migration of THP-1 monocytes was examined. Untreated medium (unconditioned medium; UCM) was used as no migration control, and recombinant human MCP-1 was used as a positive migration control. As a preliminary step, IL-6, IL-8, CXCL1, and CCL2 secretion in different conditioned media was confirmed by ELISA prior to performing migration assays. Figure 6 shows that the conditioned media CM1 and CM2, contained each of the cytokines/chemokines, while the unconditioned medium did not. In addition, conditioned media were prepared with the recombinant proteins rLlPLD1 or rLlPLD2, neutralized by specific antibody against rLlPLD1 (rLlPLD1 + rabbit polyclonal anti-rLlPLD1, CM3; and rLlPLD2 + rabbit polyclonal anti rLlPLD1, CM4) to confirm that the secretion of these cytokines/chemokines in the culture supernatant of fibroblast HFF-1 cells was a consequence of the recombinant PLD action from *L. laeta*. The pre-incubation of recombinant proteins with polyclonal antibodies raised against rLlPLD1 completely inhibited IL-6, IL-8, CXCL1, and CCL2 secretion by the fibroblasts (Figure 6), confirming that the expression and secretion of these cytokines/chemokines resulted from the effect of PLDs on the fibroblasts.

**Figure 7:**
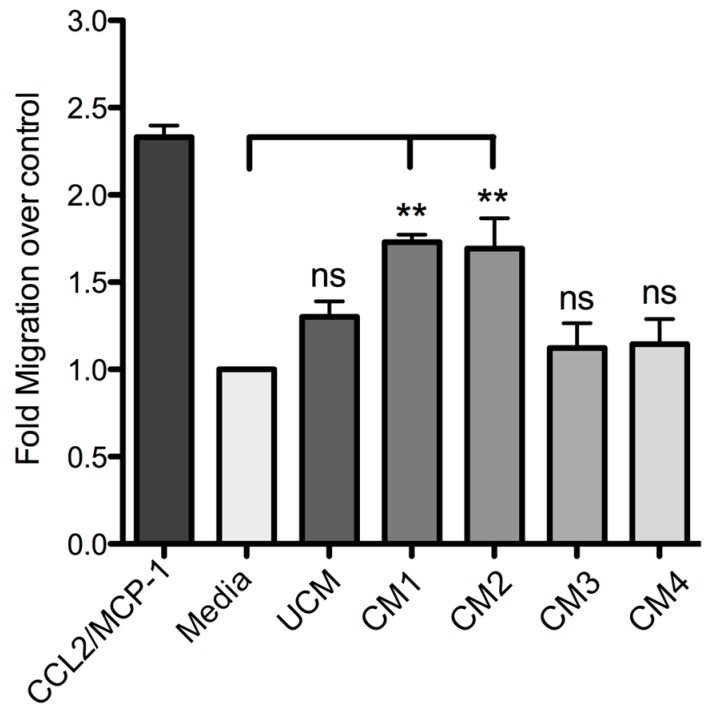
THP-1 monocyte migration assay with conditioned media from fibroblasts. THP-1 monocytes were evaluated in a migration assay in the presence of different conditioned media (CM1: rLlPLD1; CM2: rLlPLD2; CM3: neutralized rLlPLD1; and CM4: neutralized rLlPLD2). Fibroblast HFF-1 cells with no PLD treatment were also evaluated (unconditioned medium, UCM). MCP-1 at a concentration of 25 ng/mL was used as migration positive control, and RPMI 1640 serum-free medium was used as migration negative control. The migration index was calculated as x-fold increase compared to migration observed with RPMI 1640 serum-free medium control. Assays were performed in triplicate and expressed as mean ± SEM. (**) indicates *p* < 0.01 significant differences; (ns) indicates not statistically significant.

Finally, the incubation of THP-1 monocytes with the different conditioned media and subsequent assessment of migration capacity revealed that the conditioned media CM1 and CM2 could significantly induce the monocyte migration, unlike the results observed with UCM and with the conditioned media prepared with the neutralized recombinant proteins (CM3 and CM4) (Figure 7). Thus, PLDs from the *Loxosceles* venom have been demonstrated to promote the production of inflammatory mediators in human skin fibroblasts, and it has been shown that these cytokines/chemokines can act as an important chemoattractant for inflammatory cells such as monocytes.

## 3. Discussion

Loxoscelism is considered to be a public health problem in several South American countries. However, the cutaneous form of loxoscelism (CL) has been the most frequently reported (81%–84%) in Chile, with a lower incidence of systemic loxoscelism compared to CL (16%–19%) [5]. Loxoscelism in both its cutaneous and systemic clinical forms has a higher prevalence in Chile, Peru, and the Santa Catarina state of Brazil than in the rest of South America [2,26], and this is probably related with the endemic presence of the species *L. laeta*. Cutaneous loxoscelism is characterized by local signs and symptoms at the spider venom inoculation site, which involves erythema, edema, dermonecrosis, and a marked infiltration of inflammatory cells in the dermis and production of inflammatory mediators, followed by tissue necrosis [4]. These events have been documented in experimental studies, in which an increase of neutrophils is observed in the first 6 h after inoculation with *L. reclusa* venom [27,28]. Similarly, rabbits inoculated with *L. intermedia* venom displayed neutrophil accumulation in and around the blood vessels, with intense diapedesis as soon as 4 h after venom inoculation, along with a progressive accumulation of polymorphonuclear leukocytes, continuing until 5 days after animal envenomation [29]. In mice inoculated with *L. gaucho* venom, neutrophils and mononuclear cells (monocytes and lymphocytes) were the predominant inflammatory cells recruited at 24 h post-injection. Mice were also found to release inflammatory mediators such as IL-6, MCP-1, and KC (homologue to CXCL1 in humans) into serum [20]. The leukocyte incrementation and accumulation at the bite site correlate with clinical findings in patients with loxoscelism. These studies demonstrated an important leukocytosis and neutrophilia presence, increasing in patients until two weeks after poisoning [30]. In addition, some patients displayed marked leukocytosis and IL-6 and TNF-α production in plasma [31].

Monocytes are mononuclear phagocytes that can differentiate into macrophages and present antigens to drive an immune response. They have a key role as antigen-presenting cells, and in response to various stimuli, they extravasate from the bloodstream to peripheral sites, where they differentiate into macrophages and dendritic cells, contributing to the host defense as well as tissue remodeling and repair [32,33]. Considering the importance of monocytes in the inflammatory response, the present study evaluated the mechanism through which monocytes are recruited after *L. laeta* PLDs act upon human skin fibroblasts. Afterwards, the expression and secretion profiles for inflammatory cytokines and chemokines produced by fibroblasts in response to PLDs were evaluated. This study model was proposed because the main damage caused by *Loxosceles* venom is skin dermonecrosis, and cutaneous loxoscelism is the most frequent clinical presentation [5]. The PLD family is known to make up the main components of *Loxosceles* venom, and are the agents responsible for its toxic effects [4]. Different isoforms from the recombinant PLD family have been cloned and expressed, some of which have the dermonecrotic activity and others are inactive [34]. We used two PLD isoforms from *L. laeta* in our study— isoform 1 (rLlPLD1) and isoform 2 (rLlPLD2)—as previously described [25]. The latter corresponds to a catalytically-inactive isoform and does not have hemolytic activity on red blood cells. However, this isoform is highly immunogenic and confers immune-protection against dermonecrosis in rabbit models [25]. Our work has demonstrated that both recombinant PLDs from *L. laeta* were able to induce cytokine IL-6 expression and secretion, in addition to the expression and secretion of chemokines IL-8, CXCL1, and CCL2 in human skin fibroblast HFF-1 cells. This may suggest that PLD activity is not a conditioning factor for inducing the expression and secretion of inflammatory mediators. Interestingly, the effect of rLlPLD2 on the production of theses cytokines/chemokines was stronger than rLlPLD1, and its effects on the expression of the tested cytokines and chemokines was early than showed by the catalytically active isoform rLlPLD1. Although rLlPLD2 corresponds to an inactive isoform, its major effect on the expression and secretion of cytokines and inflammatory chemokines could be as a consequence of differences in its structure and interaction with the substrate at the cellular level, triggering the activation of signaling pathways that finally activates the transcriptional factor nuclear factor kappa-B (NF-κB) in a more efficient way than rLlPLD1. However, this latter must be studied further, and the mechanism by which rLlPLDs could stimulate inflammatory mediator production remains to be described. In addition, the expression of many cytokines and chemokines is regulated by the transcriptional factor NF-κB [35], so the latter may be hypothesized to have an important role in the expression of pro-inflammatory mediators activated in response to PLDs from *Loxosceles.* One interesting target could be the membrane microdomains enriched in sphingolipids (e.g., the lipid rafts), since they have an important content of sphingomyelin—one of the main substrates for PLDs from *L. laeta*. Additionally, the participation of lipid rafts has been documented in the expression of pro-inflammatory cytokines and chemokines induced by LPS and NF-κB activation [36]. Despite the many unknowns, the effect of PLD of *Loxosceles* venom on the production of inflammatory mediators and activation of NF-κB has been documented in different cell types, such as endothelial cells and fibroblasts. In this latter cell type, it has been demonstrated that human foreskin fibroblast cell line Hs68 treated with recombinant SMD from *L. reclusa* not only over-expresses IL-6, IL-8, CXCL1, and CXCL2, but also IL-1β, CCL5 (RANTES), and the transcriptional factor NF-κB [22]. The results presented in this study were consistent with the above description. However, CXCL2 was not studied here, and both IL-1β and CCL5 were not detected in the ELISArray panel screening. In addition, Human Umbilical Vein Endothelial Cells (HUVEC) and A549 epithelial cells treated with *L. deserta* venom have demonstrated increased expression and accumulative secretion of IL-8, CXCL1/GRO-α, and CCL2/MCP-1, but not CCL5 [19]; and the expression induction mechanism was via a NF-κB-dependent pathway [37]. The induction of IL-6, IL-8, and CXCL1 by recombinant PLDs from *L. laeta* on fibroblast HFF-1 cells corroborates the reported induction of cytokines and chemokines in response to the venom from other *Loxosceles* species. Importantly, our report showed the production of CCL2/MCP-1, which had not been documented to date in fibroblasts treated with recombinant PLD of *L. laeta* venom. In addition, the effect of recombinant PLD from *L. laeta* on the production of cytokines and chemokines was confirmed in the present study by demonstrating that fibroblast cultures treated with recombinant PLDs that were neutralized with specific antibodies completely abolished IL-6, IL-8, CXCL1, and CCL2 production, suggesting a direct role of PLDs in the stimulation of inflammatory mediator expression and secretion.

Additionally, the expression and secretion profile of cytokines and chemokines induced by the products of the enzymatic degradation of sphingomyelin (i.e., C1P) and lysophosphatidylcholine (i.e., LPA) were evaluated. These are known to be produced by the action of PLD from *Loxosceles* venom [9,11,17,18]. C1P has been suggested to be the inducing agent in the expression of IL-6, IL-8, CXCL1, and CXCL2 present in fibroblasts treated with recombinant *L. reclusa* PLD [22,23]. This hypothesis was challenged by van Meeteren et al. [23], who proposed that LPA rather than C1P was responsible for the induction of cytokines and chemokines expression by activating LPA receptor-mediated signaling pathways. To test this mechanism, Horta et al. [24] demonstrated that LPA mediates the release of IL-6, IL-8, CXCL1, and CXCL2, involving the participation of an LPA receptor in fibroblasts treated with either the venom of *L. similis* or recombinant PLD from *L. intermedia*. However, the direct extracellular effects of LPA were not demonstrated.

In our study, both C1P and LPA had only minor effects on the expression and secretion of the evaluated cytokines and chemokines when compared to the effects of recombinant PLD from *L. laeta*. Regardless, C1P and LPA have been documented as important inflammatory lipid mediators [38,39]. However, the role of C1P as an inflammatory mediator is contradictory. Thus, although C1P has been characterized as a pro-inflammatory molecule by cPLA2 activation and production of pro-inflammatory arachidonic acid metabolites [40], an anti-inflammatory effect has been documented depending on cell type [41]. Thus, C1P can be considered as a negative regulator of the LPS-dependent secretion for TNF-α [42], and additionally, exogenous C1P reduces the secretion of pro-inflammatory cytokines mediated by LPS in human peripheral blood mononuclear cells (PBMCs) and in HEK 293 cells expressing the TLR4 receptor through a reduction in NF-κB activation [43]. Meanwhile, LPA has the capacity to evoke and modulate immune responses by directly attracting and activating T-cells, B-cells, and macrophages, and influencing their interactions with other cell types [44]. An anti-inflammatory effect of LPA has been observed in mouse peritoneal macrophages with the activation of the ERK1/2 signaling pathway, as well as the serine/threonine phosphatases and PI3 kinase pathways, where LPA significantly inhibited the LPS-dependent secretion of TNF-α in a dose between 1 and 10 μM [45]. LPA is present in all mammalian cells and tissues, including blood, with plasma concentrations ranging from 0.1 to 1 μM, and serum concentrations that can exceed 10 μM [39]. Meanwhile, levels of C_18_-C1P in human serum have been reported at concentrations of 0.126 for males and 0.170 μM for females [46]. However, the LPA or C1P concentrations produced by *Loxosceles* PLD action under pathophysiological conditions such as cutaneous loxoscelism are unknown. Therefore, differences in the expression and secretion of cytokines and chemokines evaluated in this report cannot be ruled out as merely being a consequence of the experimental concentrations used (>10 μM), and higher concentrations of C1P and LPA could inhibit the expression and secretion of cytokines and chemokines, depending on the activation of the transcriptional factor NF-κB, which is well known as a regulator of the expression of IL-6, IL-8, CXCL1, and CCL2, as well as several other cytokines and chemokines [35]. This latter can be reaffirmed in our study, as a concentration of 10 μM induced a strong expression of the cytokines and chemokines studied in fibroblasts at 6 h of treatment, unlike what was observed with higher concentrations.

The C1P or LPA chemoattractant effect on THP-1 monocyte migration was also evaluated. In both cases, no increase in migration was observed when compared with the control. Exogenously-added C1P has been documented to be capable of inducing the cellular migration of RAW 264.7 macrophages through the participation of a C1P receptor coupled to a Gi protein [47]. This effect depends on the activation of the MEK/ERK 1-2 and PI3-K/PKB (Akt) signaling pathways and the final activation of the transcriptional factor NF-κB [48]. Similarly, C1P effects have been observed for THP-1 monocytes and pre-adipocytes 3T3 migration, only when cells were stimulated for 24 h with exogenous C1P [48]. Additionally, LPA has been demonstrated to induce cell migration of mouse embryonic fibroblasts (MEF) by activating the PI3-K/PKB (Akt) signaling pathway [49]. The discrepancies shown in our report could be due to C1P and LPA being bound to cellular receptors for efficiently inducing cell migration [48]; meanwhile, in our migration assay, the chemoattractant and cells were separated by a membrane, and cells were not stimulated before migration assay, indicating that C1P or LPA do not have a chemoattractant effect by themselves, emphasizing the importance of the interaction between C1P and its receptor. Additionally, the C1P receptor is reportedly a low affinity receptor, requiring high C1P concentrations [47]. Furthermore, high bioactive lipid concentrations could have inhibitory or adverse effects on processes such as cell proliferation; for example, LPA is reportedly able to stimulate cell proliferation of THP-1 monocytes at concentrations of 1 μM, but not at higher concentrations (10 and 20 μM), despite the induction of the expression of LPA receptors at the cellular level [50].

Finally, this study demonstrated that human skin fibroblast HFF-1 cells treated with the recombinant proteins rLlPLD1 or rLlPLD2 (conditioned medium) stimulate the migration of THP-1 monocytes. Monocytes play a pivotal role in innate immunity, since they leave the bloodstream and migrate to tissues where they differentiate into macrophage or dendritic cell populations. This recruitment is essential for effectively controlling and clearing pathogenic microorganism infections, but also contributes to the pathogenesis of inflammatory and degenerative diseases [51]. This chemotactic effect was mainly mediated by the presence of IL-8, CCL2/MCP-1, and CXCL1/GRO-α in the conditioned media; this was corroborated with fibroblast cultures treated with neutralized recombinant proteins, where the secretion of these chemoattractant mediators was completely abolished. The role of these inflammatory mediators in cell migration is well known, since these chemokines are involved in the chemotaxis of different immune cells. IL-8 and monocyte chemotactic protein-1 (MCP-1) are the most important chemokines for the recruitment of PMN cells and monocytes, respectively. In particular, CCL2 (MCP-1)—a member of the CC chemokines family—is a potent chemotactic factor for monocytes, T-lymphocytes, basophils, and natural killer (NK) lymphocytes [52]. In bacteria such as *Chlamydophila pneumoniae*, PLDs acting as a TLR4 agonist induce the expression and production of different chemokines (e.g., CCL2 in endothelial cells), and promote the migration of Th-17 cells and a Th-17-mediated immune response [53]. So, the role of the different T lymphocyte subpopulations requires further study. In addition, an evaluation of the chemotactic role of the cytokines not tested by the ELISArray panel (e.g., IL-15) during the loxoscelism inflammatory process appears to be required, since IL-15 treated monocytes have been reported to stimulate the production of chemokines such as IL-8 and CCL2/MCP-1, which favor the accumulation of inflammatory cells [54]. IL-8 (CXCL8) is considered one of the most potent neutrophil chemoattractants in inflammation, and also for basophils, T-lymphocytes, and endothelial cells [55], and CXCL1 (GRO-α) is a chemoattractant of neutrophils and endothelial cells [56]. Thus, based on our results, CCL2 can be considered as the main chemotactic mediator for monocytes, and IL-8 together with CXCL1 can be considered as the main chemotactic mediator for neutrophils in response to the PLDs of *Loxosceles.*

Our data strongly suggest that PLDs from *L. laeta* venom are the main inductors of the expression and secretion of inflammatory mediators in human skin fibroblasts during cutaneous loxoscelism, and suggest that as consequence, fibroblasts secrete inflammatory mediators which are responsible for the recruitment of monocytes to the site of damage.

## 4. Materials and Methods

### 4.1. Reagents

Dulbecco’s Modified Eagle’s Medium (DMEM), RPMI 1640, and Trypsin-EDTA (0.25%) were purchased from GIBCO (Grand Island, New York, NY, USA). Recombinant Human CCL2 (MCP-1) was purchased from BioLegend Inc (San Diego, CA, USA), LPS from *E. coli* Serotype O111:B4 (TLR grade) was obtained from Enzo Life Sciences Inc (Farmingdale, NY, USA) Ceramide 1-phosphate (C1P) from bovine brain and 1-Oleoyl-sn-Glycero-3-Phosphate (l-α-Lysophosphatidic Acid; LPA) were purchased from Sigma-Aldrich (Saint Louis, MO, USA) Stock solution of C1P was prepared as an aqueous dispersion (as liposomes) by sonicating 1 mg of C1P in 1 mL of sterile water until a clear dispersion was obtained at a final concentration of ~1.47 mM, as has been previously described [47,57]. This preparation method was preferred, since it avoids the use of organic solvent that could affect the cells in culture.

Recombinant isoforms 1 and 2 of PLD from *L. laeta* (rLlPLD1 and rLlPLD2) [25] were expressed in *E. coli* BL21 and then purified as fusion proteins with a 6xHis tag at the C-terminal by GenScript Inc. (860 Centennial Ave, Piscataway, NJ, USA), using the nucleotide sequence available in GenBank: (LlPLD1; Accession No GU121905) for a protein of 35,993 kDa with a C-6xHis tag; and (LlPLD2; Accession No GU121906) for a protein of 32,055 kDa with a C-6xHis tag. Additionally, in order to remove potential bacterial contaminant such as endotoxin from the purified recombinant proteins, an additional purification was performed by affinity chromatography using EndoTrap^®^ HD (Hyglos GmbH., Bernried, Germany) according to manufacturer’s instructions.

Polyclonal serum against rLlPLD1 was prepared in rabbits as previously described [25].

### 4.2. Cell Culture

Cells from the Human skin fibroblast HFF-1 (ATCC^®^ SCRC-1041™) and Human Monocytes THP-1 (ATCC^®^ TIB202™) cell lines were purchased from American Type Culture Collection (ATCC; Manassas, VA, USA) and cultured as indicated by the manufacturer, briefly described below.

Human skin fibroblast HFF-1 cells were cultured in Dulbecco’s Modified Eagle’s Medium (DMEM) supplemented with 15% fetal bovine serum (FBS), 100 U/mL penicillin, 100 µg/mL streptomycin, and 0.25 μg/mL Amphotericin B to made the DMEM complete growth medium, and cells were incubated at 37 °C in a humidified atmosphere containing 5% CO_2_ until confluent monolayers were obtained. When required, cells were incubated with incomplete DMEM medium (without FBS). Fibroblasts for assays were used for no more than four passages.

THP-1 monocytes were cultured in RPMI 1640 medium supplemented with 10% FBS, 0.05 mM 2-mercaptoethanol, 100 U/mL penicillin, 100 µg/mL streptomycin, and 0.25 μg/mL Amphotericin B to make the RPMI 1640 a complete growth medium. Cultures were incubated at 37 °C in a humidified atmosphere containing 5% CO_2_. The cells were split after two or three days in order to keep the cells within optimal cell density (not exceeding 1.0 × 10^6^ cells/mL).

Fibroblast HFF-1 and THP-1 monocyte cultures were stained with Trypan blue to determine cell viability prior to each experiment. Stained vs. living cells were counted under a microscope using a Neubauer modified chamber.

### 4.3. Citotoxicity Assay

Cultures of fibroblasts HFF-1 with more than 95% viability were used for cytotoxicity assays. Briefly, a cell suspension of 20,000 cells/mL in DMEM serum-free medium were placed in each well of a 96-well culture plate. Subsequently, cells were incubated for 4 h at 37 °C in a humidified atmosphere containing 5% CO_2_. Then, an initial concentration of 50 μg/mL rLlPLD1 or rLlPLD2 and concentrations of 100 μM LPA or C1P were prepared, and then two-fold dilutions were performed in DMEM medium. Untreated cells were used as 100% viability control (negative control), while cells treated with hydrogen peroxide (H_2_O_2_) 0.3% was used as a positive mortality control. Then, the culture plate was incubated at 37 °C for 24 h under an atmosphere containing 5% CO_2_, and cytotoxicity of each treatment was determined using the Cell Titer 96 Aqueous One Solution (Promega) according to the manufacturer’s instructions, under which 20 μL of reagent were dispensed to each well and incubated at 37 °C for 1 h. Subsequently, viable cells were measured at 490 nm in an Infinite M200 PRO microplate reader (Tecan Group Ltd., Männedorf, Switzerland) The percentage of viability was calculated as follows: % viability = (Abs_490nm_ sample − Abs_490nm_ blank control)/(Abs_490nm_ control 100% viability − Abs_490nm_ blank control) × 100. The inhibitory concentration 50 (IC50) for each treatment was calculated using non-linear regression on a sigmoidal curve. Assays were performed in triplicate for two independent experiments.

### 4.4. Screening of Cytokines and Chemokines Profile by ELISArray

Fibroblast HFF-1 cultures (1 × 10^5^ cells/mL) were incubated in 12-well culture plates in complete DMEM medium at 37 °C over 48 h in atmosphere containing 5% CO_2_ until confluence was reached. Subsequently, the medium was removed, and cells were washed with sterile phosphate-buffered saline (PBS). Then, the medium was replaced with fresh DMEM serum-free medium containing a concentration of 5 μg/mL rLlPLD1 or rLlPLD2, 10 μM C1P, 10 μM LPA, or 10 μg/mL LPS, and incubated for 24 h in the same conditions as before. DMEM serum-free medium was used as a control. Subsequently, cell cultures were centrifuged at 1000 × *g* for 10 min and the supernatants were recovered and evaluated for the production of cytokines or chemokines by a 12 Multi-Analyte ELISArray panel. For this, chemokines (IL8, MCP-1, RANTES, MIP-1α, MIP1-β, IP-10, I-TAC, MIG, Eotaxin, TARC, MDC, and GRO-α) were determined by Common Chemokines Multi-Analyte ELISArray kit (cat. No. MEH-009A; QIAGEN^®^), and inflammatory cytokines (IL1α, IL1β, IL2, IL4, IL6, IL8, IL10, IL12, IL17A, IFN-γ, TNF-α, and GM-CSF) were determined by Inflammatory Cytokines Multi-Analyte ELISArray (cat. No. MEH-004A; QIAGEN^®^, Germantown, MD, USA) and performed according to the manufacturer’s instructions. The absorbance of the products was measured at 450 nm in a microplate reader (Tecan, Infinite^®^ M200 PRO., Männedorf, Switzerland), and cytokine or chemokine levels were determined in relation to the absorbance value of the negative control (buffer) and compared to the positive control (containing standard of all 12 cytokines or chemokines, respectively). The presence of a determined cytokine or chemokine in culture supernatants was considered for an absorbance value over negative control. Assays were performed in duplicate.

### 4.5. Total RNA Extraction and cDNA Synthesis

Total RNA extraction from fibroblast HFF-1 cells treated with recombinant PLDs (rLlPLD1 or rLlPLD2) and C1P, LPA, or LPS, and cells without treatment (incubated with DMEM only) were carried out using the Chomczynski and Sacchi, 1987, method [58], and the TRIzol^®^ Reagent (Ambion-Life Technologies Corp., Carlsbad, CA, USA). Briefly, 6 h and 24 h fibroblast cultures, subjected to different treatments were washed twice with PBS and homogenized with 1 mL of TRIzol^®^ reagent applied directly to cell monolayers. Then, the homogenates were recovered and transferred to a 1.5 mL centrifuge tube, centrifuged at 12,000 × *g* for 20 min at 4 °C, and the aqueous phase was recovered. The RNA was precipitated by adding isopropanol, centrifuged at 12,000 × *g* for 20 min at 4 °C, and then washed with 75% ethanol. Then, total RNA was resuspended in nanopure DEPC-treated water. Total RNA integrity was evaluated by rate of spectroscopy 260/280 nm using a NanoQuant Plate Infinite^®^™ M200 PRO (Tecan, Group Ltd, Männedorf, Switzerland). Subsequently, 1 μg of total RNA was digested with DNase I Amplification Grade (Invitrogen-Life Technologies Corp., Carlsbad, CA, USA), incubated for 15 min at room temperature, and then the DNase I was inactivated by adding 25 mM EDTA to the mix, and the reaction was ended by heating the mixture at 65 °C for 10 min. Total RNA was used for cDNA synthesis using the RevertAid First Strand cDNA Synthesis kit (Thermo Fisher Scientific Inc., Waltham, MA, USA). in the presence of Oligo (dT)_18_, according to the manufacturer’s instructions. The synthesized cDNA was stored at −20 °C.

### 4.6. RT-PCR and Quantitative Real-Time RT-PCR (RT-qPCR)

RT-qPCR was performed using cDNA prepared from total RNA purified from fibroblast HFF-1 cell lysates. This cDNA (1 μL) was amplified using specific sense and antisense oligonucleotides for the human genes of IL-6 (GenBank access: NM_000600.3; amplicon of 100 bp), IL-8 (GenBank access: NM_000584.3; amplicon of 98 bp), CXCL1 (GenBank access: NM_001511.3; amplicon 128 bp), CCL2 (GenBank access: NM_002982.3; amplicon of 86 bp), and CCL5 (GenBank access: NM_002985.2; amplicon of 112 bp). Human β-actin gene (GenBank access: NM_001101.3; amplicon of 148 bp) was used as control. The primers were designed using the Primer3 software [59,60]. The sequence of primers was as follows: IL-6 (sense) 5′-CTGCTCCTGGTGTTGCCT-3′, IL-6 (antisense) 5′-CTGAAGAGGTGAGTGGCTGT-3′; IL-8 (sense) 5′-ACTGAGAGTGATTGAGAGTGG-3′, IL-8 (antisense) 5′-AGTTTTCCTTGGGGTCCAGA-3′; CXCL1 (sense) 5′-GACCAGAAGGGAGGAGGAAG-3′, CXCL1 (antisense) 5′-ATGCTCAAACACATTAGGCACA-3′; CCL2 (sense) 5′-AAGCAGAAGTGGGTTCAGGATT-3′, CCL2 (antisense) 5′-TTGGGTTGTGGAGTGAGTGTT-3′; Beta actin (sense) 5′-GTTGCGTTACACCCTTTCTTG-3′, Beta actin (antisense) 5′-CACCTTCACCGTTCCAGTTT-3’. Thermal cycling conditions were: 95 °C for 3 min, followed by 32 cycles of 95 °C for 30 s, 58 °C for 30 s, and 72 °C for 1 min, and followed by a final incubation at 72 °C for 5 min. Amplification products were separated in 1.5% agarose gel in TAE buffer and stained with EthBr, then gel images were captured in a DNR Bio-Imaging System MF-ChemiBIS 2.0. (Mahale HaHamisha, Jerusalem, Israel) using Gel-Capture software. In addition, each gene was cloned into a cloning vector pCR2.1^®^-TOPO^®^ using the TOPO TA Cloning^®^ kit (Invitrogen) and transformed into *E. coli* Novablue (EMD Chemicals-Novagen Brand., Madison, WI, USA) Then, cloned gene plasmids were purified and used to prepare a standard curve for quantitative RT-PCR (RT-qPCR) in real time. The real-time efficiencies were determined for each gene from standard curves generated from serial dilutions of plasmids sample. Then, RT-qPCR was then performed using a thermal cycler StepOnePlus™ Real-Time PCR System (Applied Biosystems., Foster City, CA, USA). Each reaction was prepared in a reaction volume of 25 μL, using 1 μL of cDNA, set primers at a concentration of 200 nM each, and reagent SYBR Green (Power SYBR^®^ Green PCR Master Mix; Applied Biosystems). Thermal cycling conditions were: denaturation step of 95 °C for 10 min, followed by 40 cycles of 95 °C for 15 s, 58 °C for 20 s, and 60 °C for 40 s. Primer specificity was checked using melting curve (60–95 °C with a heating range of 0.3 °C per second) following the manufacturer’s instructions. Differences in the mRNA expression for each gene were standardized by evaluating the human β-actin gene expression levels as an endogenous reference gene and relative to an untreated sample as calibrator using the ∆∆Ct method [61]. Results were expressed as relative gene expression using the StepOne™ Software v.2.3. (Applied Biosystems., Foster City, CA, USA)

### 4.7. ELISA Quantification of IL-6, IL-8, CXCL1/GRO-α, and CCL2/MCP-1

Fibroblasts HFF-1 cultures (1 × 10^5^ cell/mL) were treated with: rLlPLD1 or rLlPLD2 at concentrations of 1, 5, or 10 μg/mL, or C1P or LPA at 10, 30, or 50 μM, and then incubated for 24 h at 37 °C in atmosphere of 5% CO_2_. Then, the culture supernatants were collected, centrifuged at 1000× *g* for 10 min at 4 °C, and stored at −80 °C until use. Then, quantification of the different cytokines and chemokines present in culture supernatants was performed using enzyme-linked immunosorbent assays (ELISA). CCL2/MCP-1 was quantified using the LEGEND MAX™ Human MCP-1 ELISA kit (cat. No. 438807; BioLegend Inc., San Diego, CA, USA). IL-6 was quantified by using the BIOSOURCE IL-6 ELISA kit (cat. No. KAC1261; Invitrogen Corporation, Camarillo, CA, USA). CXCL1/GRO-α was quantified using the Quantikine^®^ ELISA Human CXCL1/GRO-α kit (cat. No. DGR00; R&D Systems, Minneapolis, MN, USA), and IL-8 was quantified using the Human IL-8 ELISA kit (cat. No. KHC0081; Invitrogen Corporation, Camarillo, CA, USA). In all cases, procedures were performed according to the manufacturer’s instructions, and absorbance values were measured at 450 nm for each sample and standard. Concentrations were calculated by plotting the absorbance of the standards against the standards concentration using a Four-Parameter Logistic curve fit. All assays were performed in duplicate, and results were representative of at least three independent experiments.

### 4.8. Preparation of Conditioned Media for Monocyte Migration Assay

Fibroblast HFF-1 cultures were prepared as described above and then incubated with fresh DMEM serum-free medium containing 5 μg/mL rLlPLD1 (conditioned medium 1; CM1) or 5 μg/mL rLlPLD2 (conditioned medium 2; CM2). DMEM serum-free medium was used as the unconditioned medium (UCM). In addition, further cultures of fibroblasts were prepared and treated with a conditioned medium containing neutralized recombinant protein. Briefly, a stock of 100 μg of rLlPLD1 or rLlPLD2 proteins were incubated at 37 °C for 2 h with rabbit polyclonal antibody prepared against rLlPLD1 at a dilution of 1:100. Subsequently, separate fibroblasts were incubated at 37 °C for 24 h in an atmosphere containing 5% CO_2_ with each neutralized recombinant protein at a concentration of 5 μg/mL in DMEM medium; these are referred to as conditioned medium 3 (CM3, with neutralized rLlPLD1) and conditioned medium 4 (CM4; with neutralized rLlPLD2). Finally, the supernatants of the cell cultures were recovered, centrifuged at 1000× *g* for 10 min at 4 °C, and stored at −80 °C until later analysis of THP-1 monocyte migration assays and ELISA quantification.

### 4.9. Monocyte Migration Assay

The migration of THP-1 monocyte cultures was measured using the CytoSelect™ 96-well Cell Migration Assay (Cell Biolabs, Inc.; San Diego, CA, USA), according to the manufacturer’s instructions. Briefly, THP-1 monocytes suspension (1–5 × 10^6^ cells/mL) was starved overnight prior to performing the migration assay. Subsequently, 100 μL of 2 × 10^5^ cell/mL suspension in RPMI 1640 medium were added to the upper well of the migration chamber, which had a polycarbonate membrane with a pore size diameter of 5 μm. Then, a chemoattractant (C1P or LPA) was added to the lower well of the migration chamber in 150 μL of RPMI 1640 serum-free media in concentrations of 10, 30, and 50 μM. Plates were incubated for 2, 4, or 6 h at 37 °C under a 5% CO_2_ atmosphere to allow transmigration. Cells that had transmigrated to the lower chamber were disassociated from the membrane using a detaching reagent and then lysed to be quantified using CyQuant^®^ GR Fluorescent Dye (Supplied in kit); fluorescence was read at excitation 480 nm/emission 520 nm in a Tecan Infinite M200 PRO fluorometer. Migration negative control was RPMI 1640 serum-free medium, while the migration positive control was human recombinant CCL2/MCP-1 at 25 ng/mL. The THP-1 monocyte migration was additionally evaluated using the different conditioned media as chemoattractant, which were incubated for 2, 4, or 6 h at 37 °C under a 5% CO_2_ atmosphere to allow transmigration. Cells that had transmigrated were disassociated from the membranes and quantified using CyQuant^®^ GR Fluorescent Dye at 480/520 nm. The migration index was calculated as *x*-fold increase compared to migration observed with RPMI 1640 serum-free medium control. Assays were performed in triplicate and expressed as mean ± SEM.

### 4.10. Statistical Analysis

Statistical analyses were performed using the GraphPad Prism version 5.00 for Mac OS X (GraphPad Software Inc., San Diego, CA, USA). A one-way ANOVA with Bonferroni Multiple Comparison post-hoc test was used to determine the statistical significance of differences among mean values. A statistical significance criterion significance level of *p* < 0.05 was used.

## Supplementary Materials

The following are available online at www.mdpi.com/2072-6651/9/4/125/s1, Figure S1: Cytokines ELISArray profile on human skin fibroblasts HFF-1 treated with recombinant *L. laeta* PLDs and bioactive lipids. Figure S2: RT-qPCR for expression of cytokines and chemokines in human skin fibroblasts HFF-1 cells treated with PLDs of *L. laeta* at 24 hours. Fibroblasts HFF-1 cells were incubated for 24 hours at 37 ° C in an atmosphere containing 5% CO_2_ with DMEM containing no FBS in the presence of different rLlPLD1, rLlPLD2, C1P, and LPA. A basal expression condition was evaluated in cell cultures treated with only DMEM medium. cDNA was synthesized from mRNA taken from the supernatant in each experiment and amplified by RT-qPCR. The human β-actin gene was used as the reference gene. Differences in the relative expression of human genes IL-6, IL-8, CXCL1 and CCL2 mRNA expression for each gene were standardized using the human β-actin gene expression levels as a reference gene, and results were presented as the average relative expression over control ± SEM of two experiments performed in duplicate.
