# Supplementary material for: Phospholipase D from Loxosceles laeta Spider Venom Induces IL-6, IL-8, CXCL1/GRO-α, and CCL2/MCP-1 Production in Human Skin Fibroblasts and Stimulates Monocytes Migration
Source: Toxins (Basel). 2017 Apr 5;9(4):125. doi: 10.3390/toxins9040125 (PMC5408199; doi:10.3390/toxins9040125)
Supplement: Supplementary file 1 [file toxins-09-00125-s001.pdf]

Supplementary Materials: Phospholipase D from *Loxosceles laeta* Spider Venom Induces IL-6, IL-8, CXCL1/GRO-*α*, and CCL2/MCP-1 Production in Human Skin Fibroblasts and Stimulates Monocytes Migration

José M. Rojas, Tomás Arán-Sekul, Emmanuel Cortés, Romina Jaldín, Kely Ordenes, Patricio R. Orrego, Jorge González, Jorge E. Araya and Alejandro Catalán

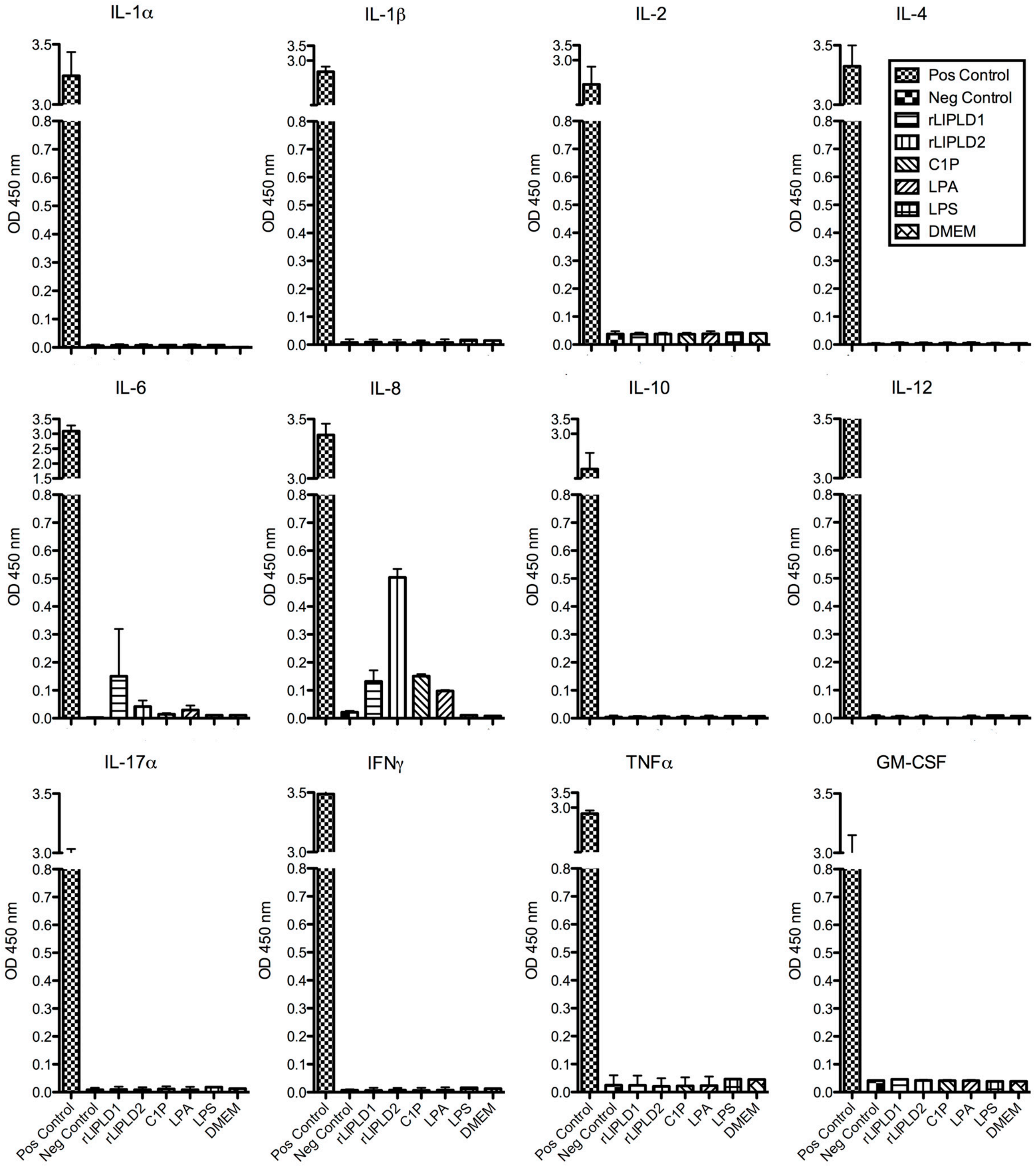

Figure S1. Cytokine ELISArray profiles of human skin fibroblasts HFF-1 cells. Fibroblasts HFF-1 (1 x 10^5 cells/mL) cultures were treated with 5 μg/mL rLlPLD1 or rLlPLD2, 10 μM C1P, 10 μM LPA or 10 μg/mL LPS and incubated for 24 h at 37 °C in an atmosphere containing 5% CO_2. DMEM medium

without FBS was used as control. Culture supernatants were recovered and used for cytokine screening in a Inflammatory Cytokines Multi-Analyte ELISArray kit (QIAGEN) panel for the detection of IL1α; IL1β; IL2; IL4; IL6; IL8; IL10; IL12; IL17A; IFN-γ; TNF-α; and GM-CSF, according to manufacturer's instructions. Absorbance at 450 nm was measured in a micro-plate reader and the cytokines levels were determined in relation to the absorbance value of the negative control (assay buffer) and compared to the positive control (containing standard of all 12 cytokines). Presence of cytokine in culture supernatants was considered for absorbance values over negative control. Results were expressed as mean ± SEM of two experiments performed independently.

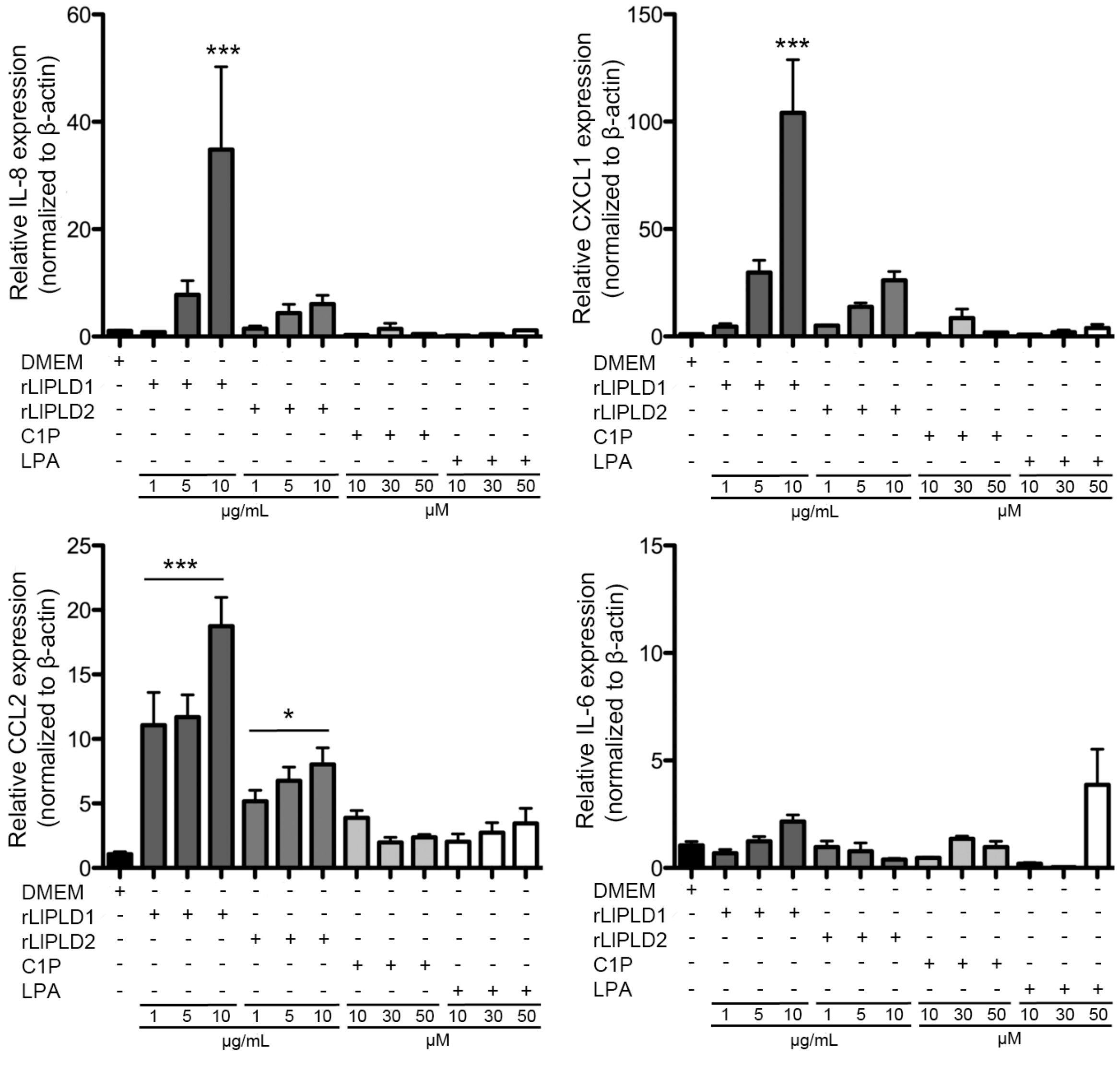

Figure S2. RT-qPCR for expression of cytokines and chemokines in human skin fibroblasts HFF-1 cells treated with PLDs of *L. laeta* at 24 hours. Fibroblasts HFF-1 cells were incubated for 24 hours at 37 ° C in an atmosphere containing 5% CO_2 with DMEM containing no FBS in the presence of different rLlPLD1, rLlPLD2, C1P, and LPA. A basal expression condition was evaluated in cell cultures treated with only DMEM medium. cDNA was synthesized from mRNA taken from the supernatant in each experiment and amplified by RT-qPCR. The human β-actin gene was used as the reference gene. Differences in the relative expression of human genes IL-6, IL-8, CXCL1 and CCL2 mRNA expression

for each gene were standardized using the human β-actin gene expression levels as a reference gene, and results were presented as the average relative expression over control ± SEM of two experiments performed in duplicate.
